# Different doses of galcanezumab versus placebo in patients with migraine and cluster headache: a meta-analysis of randomized controlled trials

**DOI:** 10.1186/s10194-020-1085-x

**Published:** 2020-02-11

**Authors:** Yanbo Yang, Zilan Wang, Bixi Gao, He Xuan, Yun Zhu, Zhouqing Chen, Zhong Wang

**Affiliations:** 1 Department of Neurosurgery & Brain and Nerve Research Laboratory, The First Affiliated Hospital of Soochow University, 188 Shizi Street, Suzhou, Jiangsu Province, 215006 China; 2 Department of Cardiology, The First Affiliated Hospital of Soochow University, Suzhou, Jiangsu Province, 215006 China

**Keywords:** Migraine, Cluster headache, Galcanezumab, Meta-analysis

## Abstract

**Background:**

Galcanezumab is a novel monoclonal antibody that target to calcitonin gene-related peptide (CGRP). It has been tested for the preventive treatment of migraine and episodic cluster headache by multiple randomized clinical trials (RCTs) and have been found to reduce headache frequency.

**Methods:**

We systematically searched PubMed and Embase on Cochrane Central Register of Controlled Trials (CENTRAL) from the earliest date to August 1, 2019. Relative risk (RR) and weighted mean difference (WMD) were used to evaluate clinical outcomes.

**Results:**

Seven studies were pooled with 3889 patients. Subcutaneous injection of Galcanezumab at 120 mg, 240 mg leads to a statistically significant response rate for the treatment of migraine compared with placebo (120 mg: RR = 1.51; 95% CI, 1.33 to 1.70; *P* < 0.001; 240 mg: RR = 1.58; 95% CI, 1.43 to 1.76; *P* < 0.001). Among them, 120 mg group has the same treatment efficacy with 240 mg group (50% response: RR = 1.06; 95% CI, 0.92 to 1.22; *P* = 0.425; 75% response: RR = 1.07; 95% CI, 0.94 to 1.23; *P* = 0.301; 100% response; RR = 1.06; 95% CI, 0.81 to 1.37; *P* = 0.682; MHD: RR = − 0.08; 95% CI, − 0.55 to − 0.40; *P* = 0.748) while related to a lower risk for adverse events for the treatment of migraine (120 mg RR = 1.06; 95% CI, 0.99 to 1.14; *P* = 0.084; 240 mg: RR = 1.17; 95% CI, 1.09 to 1.25; *P* < 0.001). 300 mg per month galcanezumab is effective for the prevention of episodic cluster headache measured by at least 50% reduction of cluster headache frequency at week 3 (RR = 1.36; 95% CI, 1.00–1.84; *P* = 0.048).

**Conclusions:**

Use of galcanezumab is related to a significantly reduced monthly headache frequency compared with placebo for the treatment of migraine and episodic cluster headache, 120 mg has the same treatment efficacy with 240 mg group while related to a lower risk for adverse effects for the treatment of migraine. 300 mg per month galcanezumab is effective for the prevention of episodic cluster headache with no significantly increased adverse events.

## Background

Migraine and cluster headache are disabling neurologic disorder characterized by severe and repetitive headache attacks. As proposed by the International Headache society, migraine is defined as headache attacks lasting 4–72 h that are accompanied by nausea, photophobia or phonophobia, while cluster headache is a strictly unilateral headache that lasts for 15–180 min and is commonly combined with symptoms that related with parasympathetic activation [1]. Both migraine and cluster headache can lead to severe psychological disturbance [2, 3] and have caused a huge economic burden [4, 5].

Galcanezumab is a monoclonal antibody that targets to the calcitonin gene-related peptide (CGRP). Increased serum CGRP level is observed after migraine and cluster headache attack in both human and animal models [6, 7]. Trigeminovascular system—a pain modulating center—can release CGRP upon activation [8]. CGRP will, induce potent vasodilatory effects on cerebral arteries [9], modulate the sensitivity of nociceptive trigeminal neurons [10] and subsequently trigger migraine and cluster headache attacks. This process is considered as an initiative step in the pathogenesis of both migraine and cluster headache [11–13]. For decades, CGRP is considered a promising target for treatment of migraine and cluster headache. Previous clinical trials have supported the use of CGRP monoclonal antibodies as a preventive treatment for migraine and episodic cluster headache [14–20]. The development of drugs that targets to CGRP has become a successful translation from bench to clinic [10].

Systemic reviews have confirmed the effectiveness and safety of CGRP monoclonal antibodies for the treatment of migraine and cluster headache [21, 22]. However, until now, no study is conducted to systemically analyze the efficacy and safety profiles of galcanezumab across different doses, therefore, we conducted a meta-analysis to evaluate the dose-related efficacy and tolerability of galcanezumab for the treatment of migraine and cluster headache.

## Methods

A meta-analysis was conducted in accordance with the preferred reporting items for systematic reviews and meta-analyses (PRISMA) guidelines [23].

### Search strategy

Original researches in the PUBMED, EMBASE database and Cochrane Library database were searched up to August 1, 2019 for relevant published articles. The following search terms were used: galcanezumab, LY2951742, migraine and cluster headache. The reference lists of the relevant articles were all screened to avoid omissions.

### Study selection

All of the studies included in this meta-analysis (1) were all randomized clinical trials (RCTs); (2) enrolled participants with migraine or cluster headache (3) used galcanezumab as intervention; (4) enrolled over 100 participants.

### Data extraction

All the data were extracted independently by 3 investigators (YY, ZW and ZC) and any disagreements were settled through discussion. The extracted data include (1) literature information (title, author, publication time, sample size, etc.); (2) characteristic of the object of the study (age, BMI, smoking status, headache attack status before study); (3) content of the expose or interfere (experiment doses in different groups); (4) outcome data including migraine headache days (MHD), 50%, 75%, 100% response rate, which was defined as a reduction of the frequency of headache attacks by at least given percentage, treatment-emergent adverse events (TEAE), serious adverse events (SAE) and number of patients discontinue. We chose 50% response rate at the end of each trial as the primary endpoint for migraine, 50% response rate at week 3 for cluster headache.

### Statistical analysis

In this study, weighted mean differences (WMD) and relative risk (RR) with their 95% confidence intervals (CIs) were used to evaluate the effect of galcanezumab on migraine or cluster headache. The heterogeneity between the included studies was evaluated with *I*^*2*^ and *p*-value. When the *I*^*2*^ > 50% or *p*-value< 0.05, the data would be regarded as showing conspicuous heterogeneity. Random-effects model was applied to analyze the data. In addition, the researchers divided the subjects into different subgroups according to the dose and time of drug use, and made a subgroup analysis. Details of the subgroup analysis will be described later in the article. STATA 12.0 was used to conduct the analysis.

### Risk of Bias

The risk of bias plot in individual studies was created using the Review Manager 5.2 software. For assessing the risk of bias of RCTs, we applied uniform criteria of the Cochrane collaboration, which included: selection bias, performance bias, detection bias, attrition bias, reporting bias, and other potential biases.

## Results

### Search results and study characteristics

**Fig. 1:**
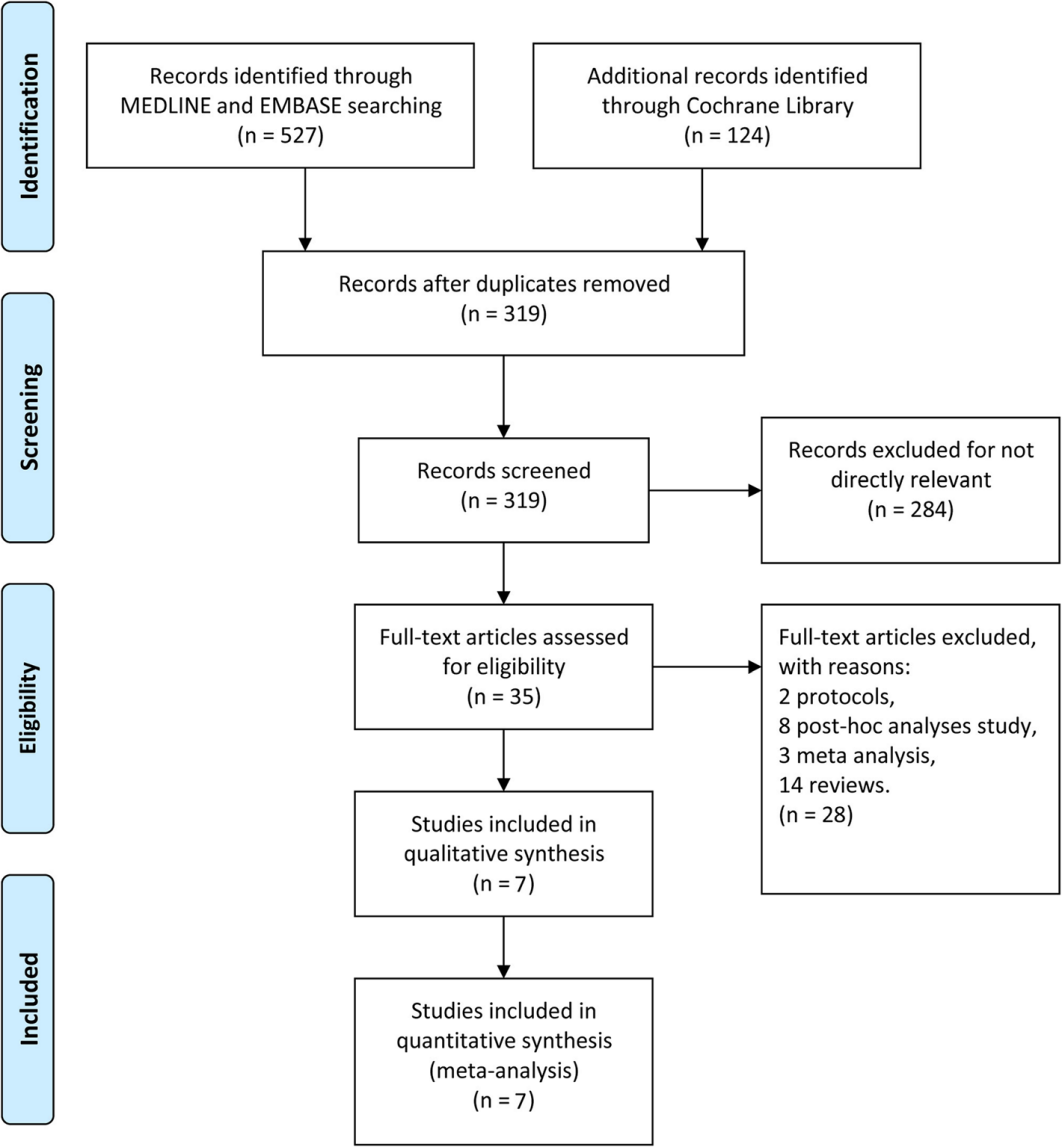
The study search, selection, and inclusion process

We systematically searched articles published before August 1, 2019 and identified 651 articles in total that were related to this topic. After removing the duplicate reports, there were 319 reports remaining. Among these, 7 studies were found to be directly related to our study, but one ongoing clinical trial on chronic cluster headache (NCT02438826) was excluded because the trial did not reach its primary endpoint. Lastly, 3889 patients from 6 multi-centered double blinded trials [14–19], one open-labeled clinical trial [20] were pooled. Among them, 6 trails focused on chronic and/or episodic migraine, and one trial on episodic cluster headache. Study selection process was plotted in Fig. 1. Details of the study characteristics are shown in Table 1.

**Table 1:** Characteristics of the Included Studies and Outcome Events

Trials Phases Publication Center	Inclusion Criteria	Exclusion Criteria	Condition	group	Frequency
Oakes, 2018(NCT02163993) [14] Phase2b Cephalalgia Multicenter	Aged 18–65 years A diagnosis of episodic migraine for at least 1 year prior Monthly frequency of 4–14 MHD	Failure to respond to three or more adequately dosed effective migraine prevention treatments Prior exposure to any CGRP antibody, any antibody to the CGRP receptor, or antibody to NGF History of migraine subtypes^a^	Episodic migraine	galcanezumab 5 mg galcanezumab 50 mg galcanezumab 120 mg galcanezumab 300 mg placebo	Once a month for 3 months
Stauffer, 2018(NCT02614183) [15] Phase 3 JAMA Neurology Multicenter	Aged 18–65 years A diagnosis of episodic migraine for at least 1 year prior Monthly frequency of 4–14 MHD	Failure to respond to three or more classes of migraine preventive treatments Prior exposure to any CGRP antibody Having taken a therapeutic antibody in the past 12 months Receiving preventive migraine medication within 30 days History of persistent daily headache, cluster headache or migraine subtypes	Episodic migraine	galcanezumab 120 mg (with 240-mg loading dose) galcanezumab 240 mg placebo	Once a month for 6 months
Skljarevski, 2018(NCT02614196) [16] Phase 3 Cephalalgia Multicenter	Aged 18–65 years A diagnosis of episodic migraine for at least 1 year prior Monthly frequency of 4–14 MHD and at least two migraine attacks during the baseline period	Failure to respond to three or more classes of migraine preventive treatments Prior exposure to any CGRP antibody Having taken a therapeutic antibody in the past 12 months Using opioids or barbiturates more than twice per month Known hypersensitivity to multiple drugs	Episodic migraine	galcanezumab 120 mg galcanezumab 240 mg placebo	Once a month for 6 months
Dodick, 2014 (NCT01625988) [19] Phase 2 Lancet Neurol Multicenter	Aged 18–65 years A diagnosis of migraine as defined by IHS ICHD-II for at least 1 year prior Monthly frequency of 4–14 MHD	Failure to respond to three or more adequately dosed approved migraine prevention treatments Receiving preventive migraine medication within 30 days, including receiving botulinum toxin within 4 months History of chronic migraine or migraine subtypes Patients with at least 15 headache days per 28-day period	Episodic migraine	galcanezumab 150 mg placebo	Once every 2 weeks for 12 weeks
Trials Phases Publication Center	Inclusion Criteria	Exclusion Criteria	Condition	group	Frequency
Detke, 2018(NCT02614261) [17] Phase 3 Neurology Multicenter	Aged 18–65 years A diagnosis of chronic migraine for at least 1 year prior A history of at least 15 headache days per month, of which at least 8 were migraine and a history of at least 1 headache-free day per month for the past 3 months	Failure to respond to three or more classes of migraine preventive treatments Prior exposure to any CGRP antibody Having taken a therapeutic antibody in the past 12 months Persistent daily headache, cluster headache, head or neck trauma within the past 6 months, possible posttraumatic headache, or primary headache other than chronic migraine Receiving preventive migraine medication within 30 days, including receiving botulinum toxin within 4 months	Chronic migraine	galcanezumab 120 mg (with 240-mg loading dose) galcanezumab 240 mg placebo	Once a month for 3 months
Camporeale, 2018(NCT02614287) [20] Phase 3 BMC Neurology Multi-center	Aged 18–65 years A diagnosis of migraine for at least 1 year prior A history of 4 or more MHD per month on average for the past 3 months and a history of at least 1 headache-free day per month for the past 3 months	Failure to respond to three or more classes of migraine preventive treatments Prior exposure to galcanezumab or any other CGRP antibody Having taken a therapeutic antibody in the past 12 months Current treatment with preventive migraine medication	Chronic and episodic migraine	galcanezumab 120 mg (with 240-mg loading dose) galcanezumab 240 mg	Once a month for 12 months
Goadsby, 2019(NCT02397473) [18] Phase 3 N Engl J Med Multicenter	Aged 18–65 years A diagnosis of cluster headache Frequency of at least one attack every other day, at least four total attacks, and no more than eight attacks per day during a baseline assessment A history of cluster headache periods lasting at least 6 weeks Participants are able to distinguish cluster headache attacks from other headaches	Prior exposure to any CGRP antibody, antibody to the CGRP receptor or antibody to NGF Concurrent use of other therapeutic monoclonal antibodies Having another distinct trigeminal autonomic cephalalgia or a history of migraine variants that could have been due to cerebral ischemia	Episodic cluster headache	galcanezumab 300 mg placebo	Once a month for 2 months

*MHD* Migraine Headache Days, *CGRP* calcitonin-gene-related peptide, *NGF* nerve growth factor;

^a^Migraine subtypes including hemiplegic migraine, ophthalmoplegic migraine, and basilar-type migraine

### Quality assessment

**Fig. 2:**
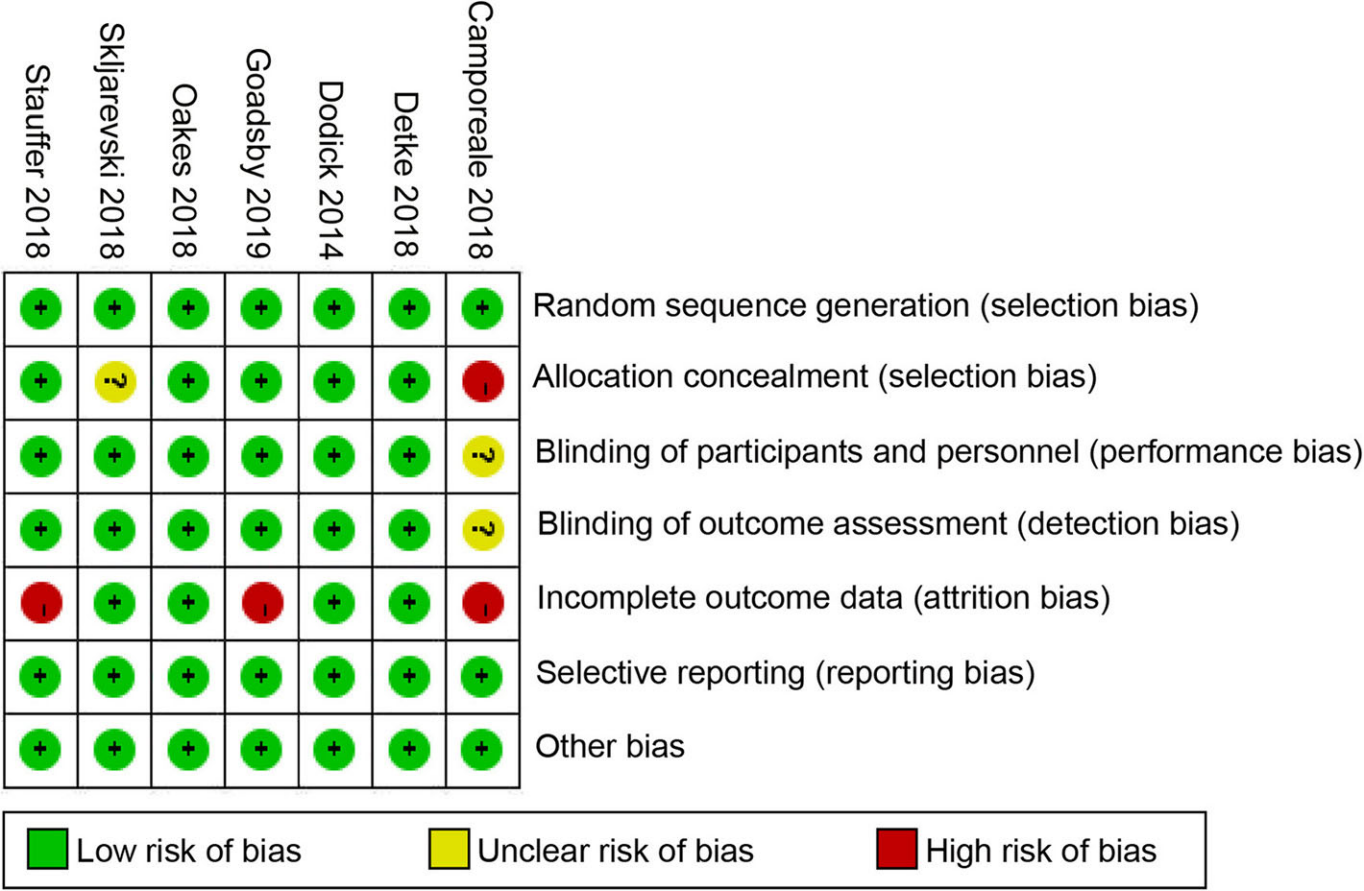
Summary table for potential bias analysis for included study

Three of the seven studies are at high risk of attrition bias [15, 18, 20] which can be explained by a relatively high discontinue rate related to prolonged experiment period [15, 20] and high proportion of discontinue in placebo group due to lack of efficacy [18]. One study is at high risk of selection bias [20]. We conducted a sensitivity test to evaluate potential bias of the studies involved in our meta-analysis. Of the seven included studies, all the studies were at low risk of publication bias. There were six double-blind and one open-label studies, with the latter deemed higher risk of bias but there was no evidence of bias for the primary outcome (Fig. 2).

### Efficacy

**Fig. 3:**
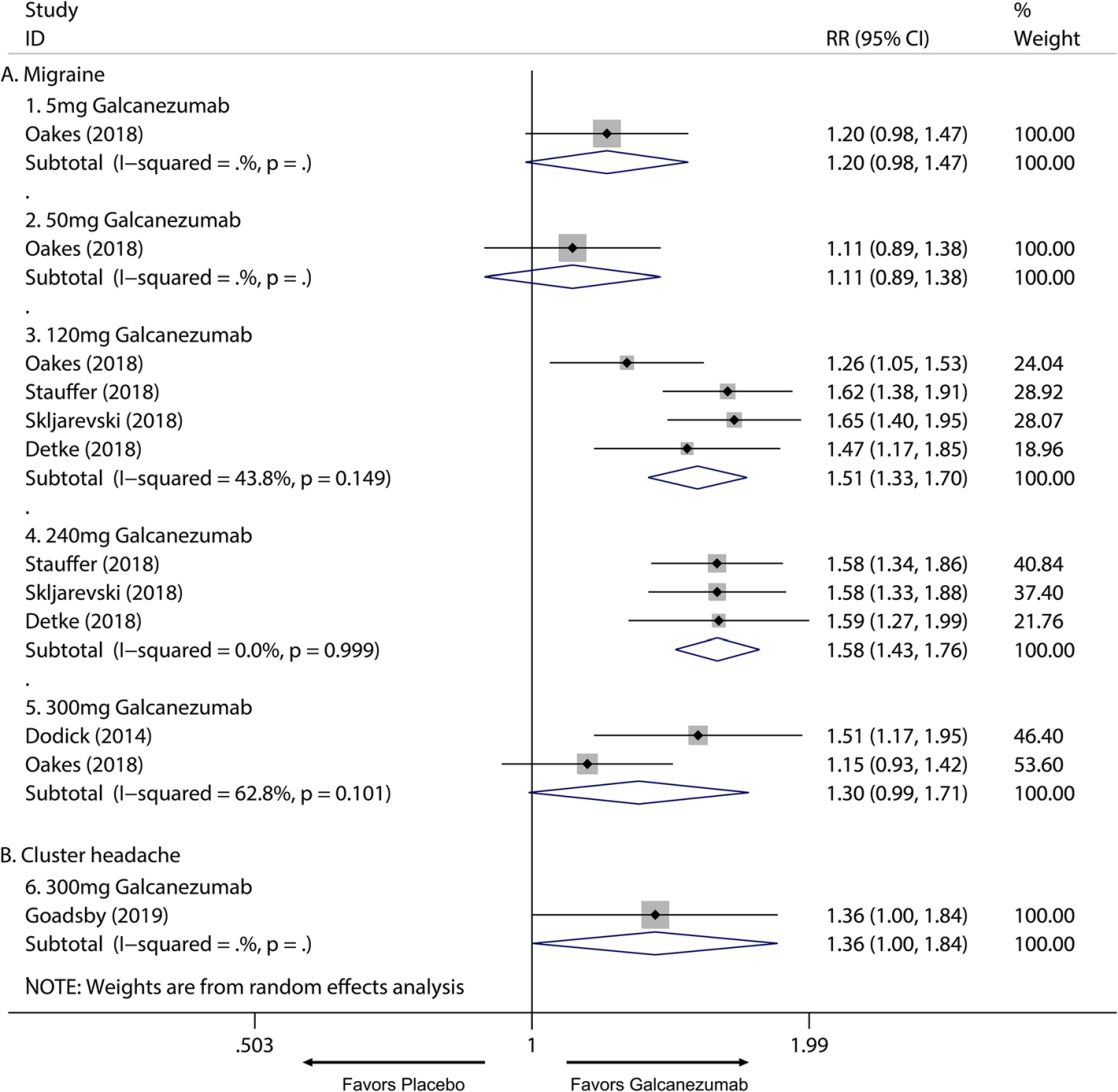
The pooled relative risk of the 50% response rate (defined as at least 50% reduction of headache attack frequency from baseline) in different treatment doses compared with placebo, the diamond indicates the estimated relative risk with 95% confidence interval for the pooled patients. *RR* relative risk

**Fig. 4:**
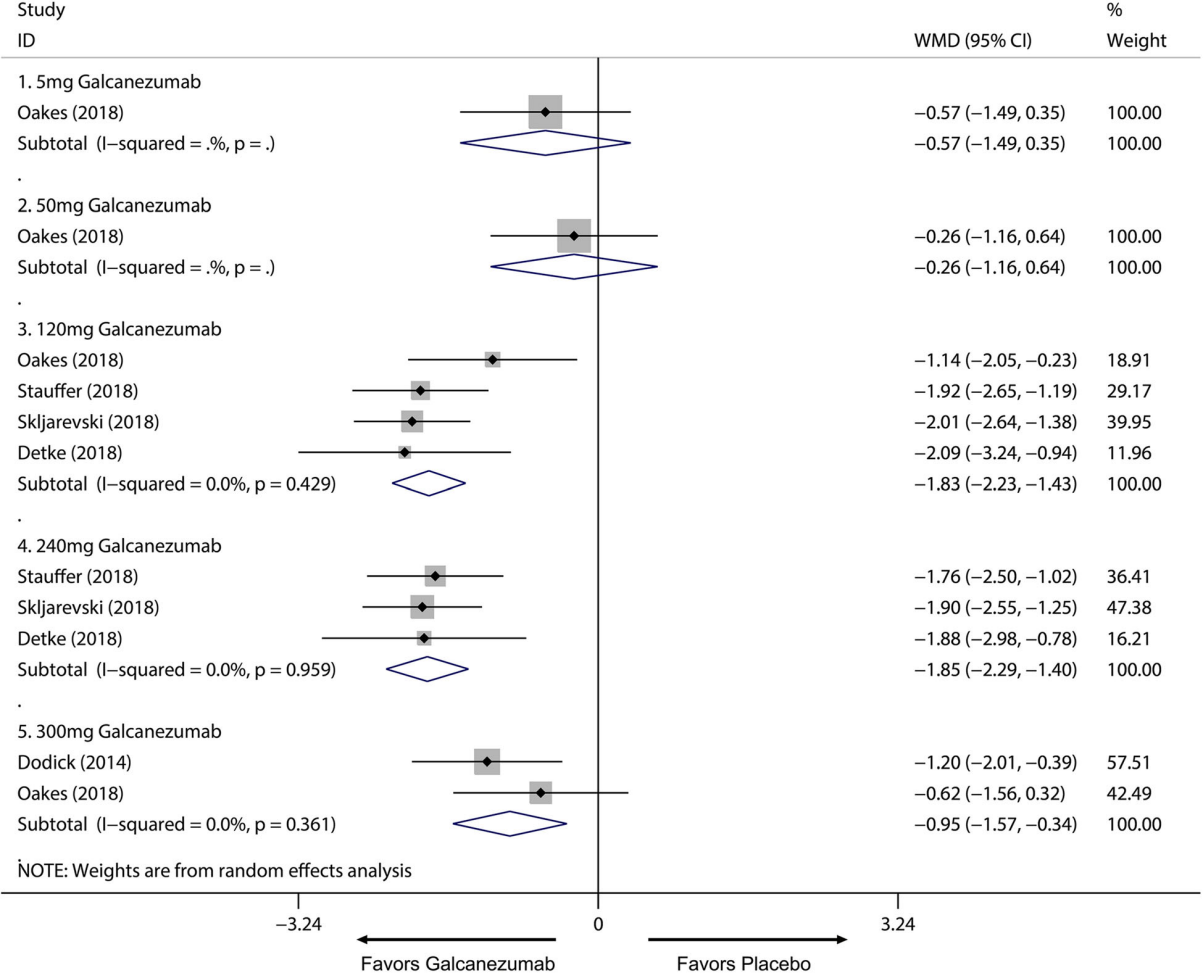
The pooled weighted mean differences of the reduction of MHDs in treatment group compared with placebo in different doses, the diamond indicates the estimated relative risk with 95% confidence interval for the pooled patients. *WMD* weighted mean differences

**Fig. 5:**
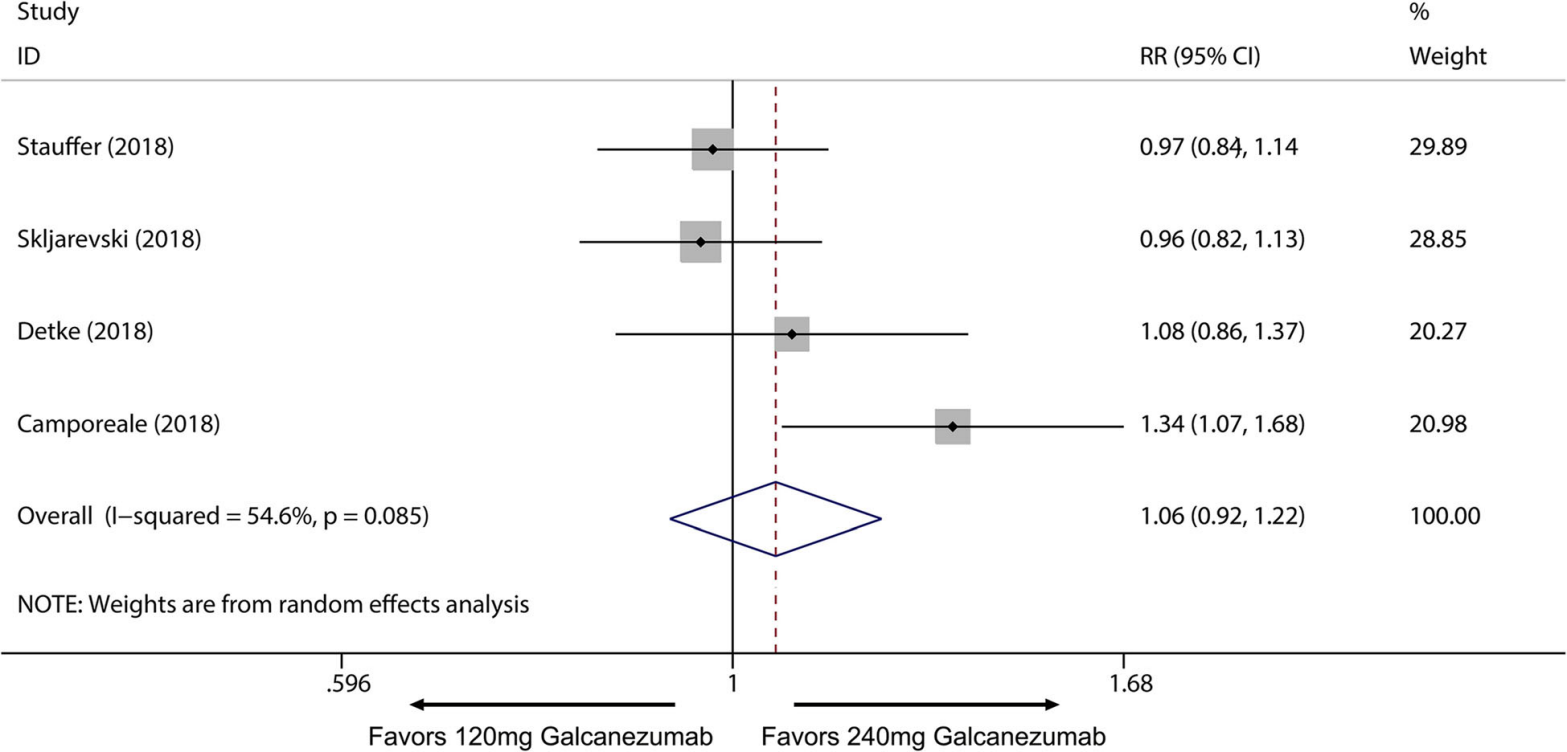
Comparison of 50% response rate between 120 mg and 240 mg galcanezumab. *RR* relative risk

**Fig. 6:**
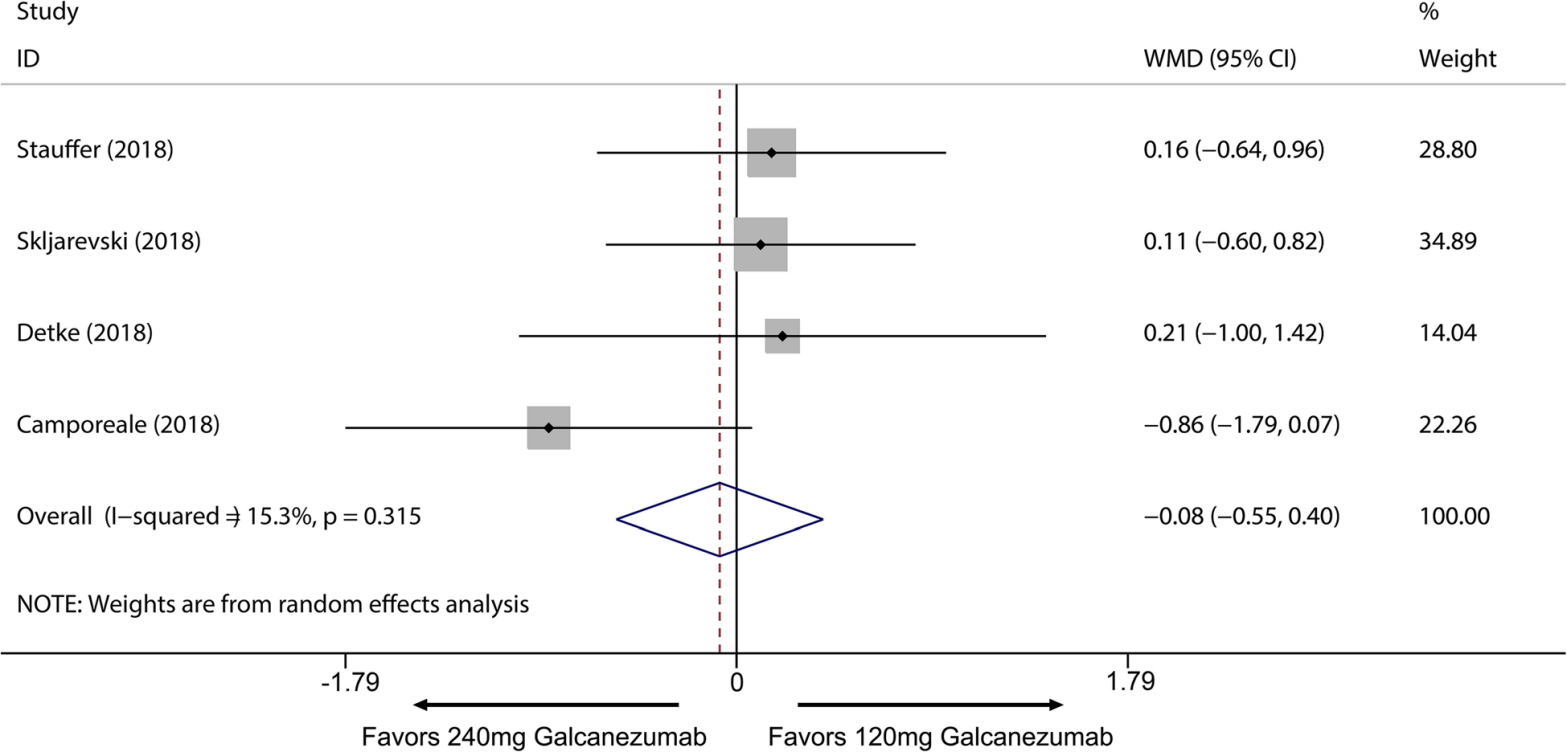
Comparison of MHDs reduction between 120 mg and 240 mg galcanezumab. *WMD* weighted mean differences

Overall, the use of galcanezumab was associated with significant increased response rate compared with placebo (Additional file 1: Supplement 1). The sensitivity analysis did not substantively alter the overall result (Additional file 1: Supplement 2). When measured by 50% response rate across different dosage subgroup, subcutaneous injection of galcanezumab with 120 mg and 240 mg doses were related to a significantly increased 50% response rate compared with placebo group for the treatment of migraine (120 mg: RR = 1.51; 95% CI, 1.33 to 1.70; *P* < 0.001; 240 mg: RR = 1.58; 95% CI, 1.43 to 1.76; *P* < 0.001) (Fig. 3a) and 5 mg and 50 mg doses did not related to increased 50% response rate compared with placebo (5 mg: RR = 1.20; 95% CI, 0.98 to 1.47; *P* = 0.071; 50 mg: RR = 1.11; 95% CI, 0.89 to 1.38; *P* = 0.367) (Fig. 3a). 75% and 100% response rate of 120 mg and 240 mg subgroup also had proved this dose-response relationship for the treatment of migraine (Additional file 1: Supplement 3 and Additional file 1: Supplement 4). For MHDs, 120 mg, 240 mg and 300 mg galcanezumab were associated with significantly reduced MHDs (120 mg: WMD = − 1.83; 95% CI, − 2.23 to − 1.43; *P* < 0.001; 240 mg: WMD = − 1.85; 95% CI, − 2.29 to − 1.40; *P* < 0.001; 300 mg: WMD = − 0.95, 95% CI, − 1.57 to − 0.34; *P* = 0.002) while 5 mg and 50 mg had no significant treatment effect (5 mg: WMD = − 0.57; 95% CI, − 1.49 to 0.35; *P* = 0.225; 50 mg: WMD = − 0.26; 95% CI, − 1.16 to 0.64; *P* = 0.572) (Fig. 4). For comparing efficacy of galcanezumab between 120 mg and 240 mg for treatment of migraine, no significant differences in 50%, 75%, 100% response rates and MHDs reduction between 120 mg and 240 mg group were found (50% response: RR = 1.06; 95% CI, 0.92 to 1.22; *P* = 0.425; 75% response: RR = 1.07; 95% CI, 0.94 to 1.23; *P* = 0.301; 100% response; RR = 1.06; 95% CI, 0.81 to 1.37; *P* = 0.682; MHD: RR = − 0.08; 95% CI, − 0.55 to − 0.40; *P* = 0.748) (Fig. 5; Additional file 1: Supplement 5; Additional file 1: Supplement 6; Fig. 6). Sensitivity analysis showed that all of the results were regarded stable (Additional file 1: Supplement 7). For the efficacy of galcanezumab across different timepoints, galcanezumab was effective in reducing MHDs and improve 50% response rate in both patients with migraine at 120 mg, 240 mg and 300 mg dose in any timepoint of interest (Table 2). For the treatment of cluster headache, 300 mg was effective against episodic cluster headache measured by 50% response rate at week 3 compared with placebo. (RR = 1.36; 95% CI, 1.00 to 1.84; *P* = 0.048) (Fig. 3b).

**Table 2:** Subgroup analysis of efficacy and safety outcome of galcanezumab for the treatment of migraine

	Efficacy				Safety	
	50% ≥ response		MHD		TEAE	
	RR (95% CI)	*P* value	WMD (95% CI)	*P* value	RR (95% CI)	*P* value
1. **120 mg Galcanezumab**						
3 months	1.469 (1.166, 1.850)	0.006	−2.090 (−3.237, − 0.943)	0.000	1.144 (1.003, 1.304)	0.045
6 months	1.513 (1.286, 1.779)	0.013	−1.774 (−2.256, − 1.291)	0.000	1.033 (0.950, 1.123)	0.443
2. **240 mg Galcanezumab**						
3 months	1.589 (1.270, 1.987)	0.013	−1.880 (−2.983, −0.777)	0.001	1.155 (1.014, 1.316)	0.030
6 months	1.582 (1.406, 1.780)	0.000	−1.839 (−2.324, − 1.354)	0.000	1.170 (1.082, 1.266)	0.000
3. **300 mg Galcanezumab**						
1 month	N/A	N/A	N/A	N/A	1.286 (0.788, 2.097)	0.314
3 months	1.509 (1.166, 1.954)	0.000	−1.200 (−2.012, −0.388)	0.004	1.087 (0.927, 1.275)	0.302
6 months	1.147 (0.928, 1.418)	0.000	−0.620 (−1.565, 0.325)	0.198	0.935 (0.693, 1.261)	0.659

*RR* Relative Risk, *CI* Confidence Interval, *N/A* Not Applicable

### Tolerance, adverse effect, and adherence

Of all the 2133 patients receiving galcanezumab, no deaths occurred during the experiment period. The most frequently reported adverse effects were injection site pain and upper respiratory infections. Subsequently, a subgroup analysis was performed to examine the difference in TEAEs and SAEs on effective treatment doses for migraine. We found 120 mg group had no significant difference in TEAEs compared with placebo group (RR = 1.06; 95% CI, 0.99 to 1.14; *P* = 0.084) (Fig. 7.1), and a higher frequency of adverse events in the 240 mg group than placebo group was identified (RR = 1.17; 95% CI, 1.09 to 1.25; *P* < 0.001) (Fig. 7.2). Moreover, 120 mg and 240 mg group had a significant increase in serious adverse effects frequency than placebo group (120 mg: RR = 2.10; 95% CI, 1.02 to 4.36; *P* = 0.045; 240 mg: RR = 2.75; 95% CI, 1.38 to 5.47; *P* = 0.004) (Fig. 8). For the comparing adverse events of galcanezumab between 120 mg and 240 mg, 120 mg galcanezumab is related to decreased risks for TEAEs and AEs compared with 240 mg galcanezumab (Fig. 9). For safety of galcanezumab at effective treatment dose (300 mg) for episodic cluster headache, the pooled data showed 300 mg galcanezumab is not related with significant increased risks for TEAEs and SAEs compared with placebo (TEAEs: RR = 0.93; 95% CI, 0.93 to 1.22; *P* = 0.340; SAEs: RR = 0.79; 95% CI, 0.25 to 2.47; *P* = 0.688) (Fig. 10).

**Fig. 7:**
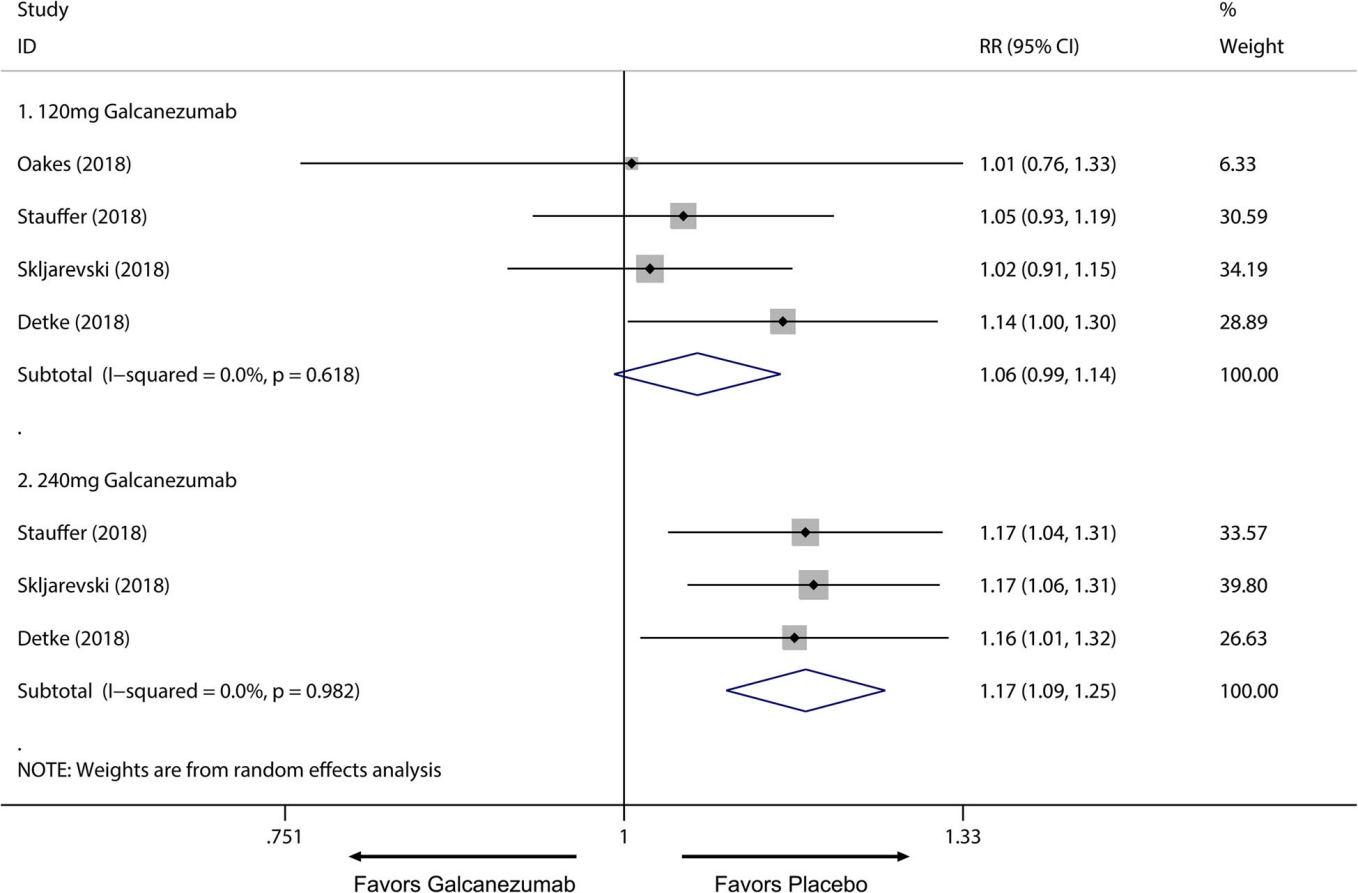
Pooled TEAEs relative risk in 120 mg and 240 mg galcanezumab group. *RR* relative risk

**Fig. 8:**
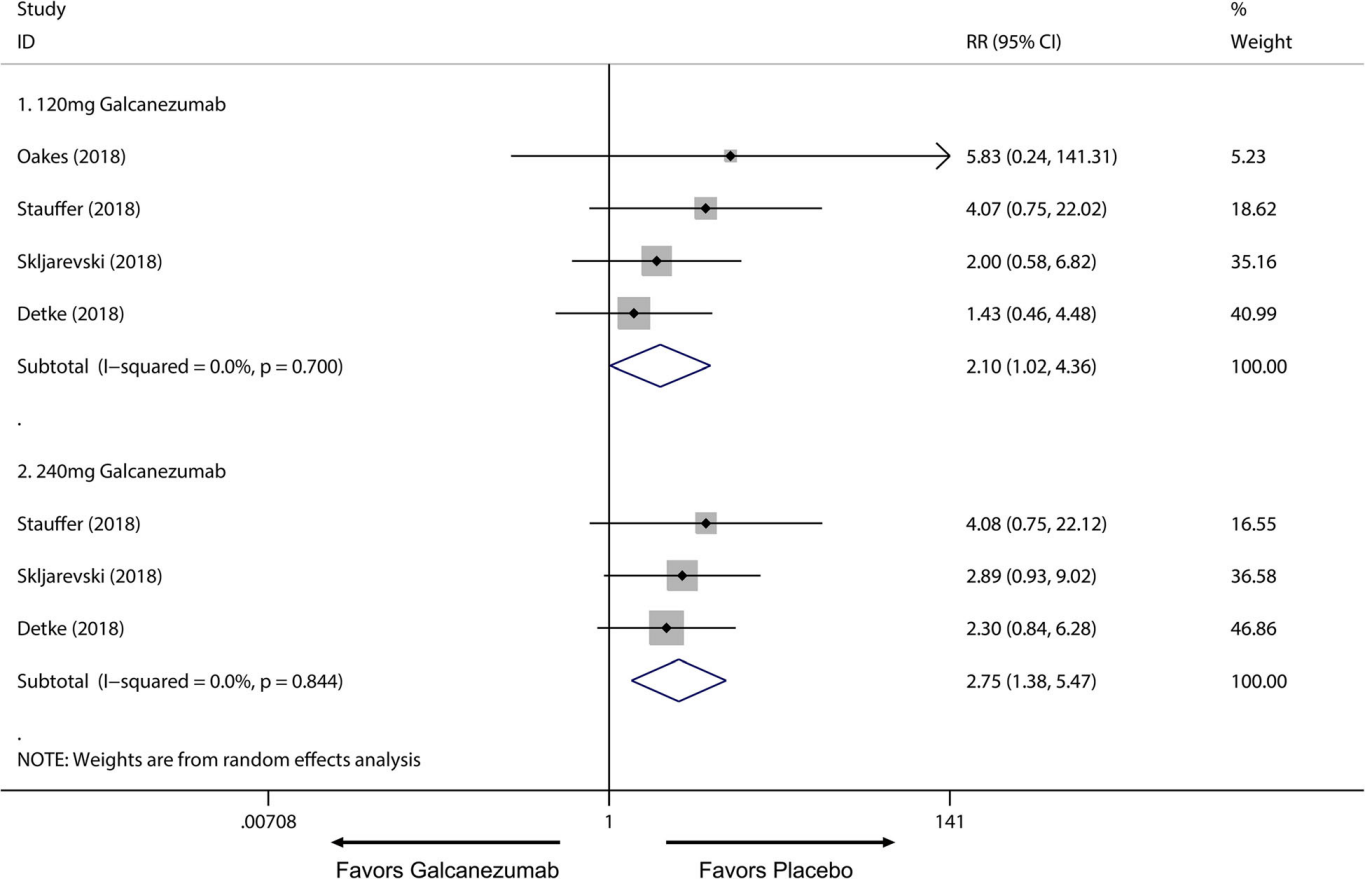
Pooled SAEs relative risk in 120 mg and 240 mg galcanezumab group. *RR* relative risk

**Fig. 9:**
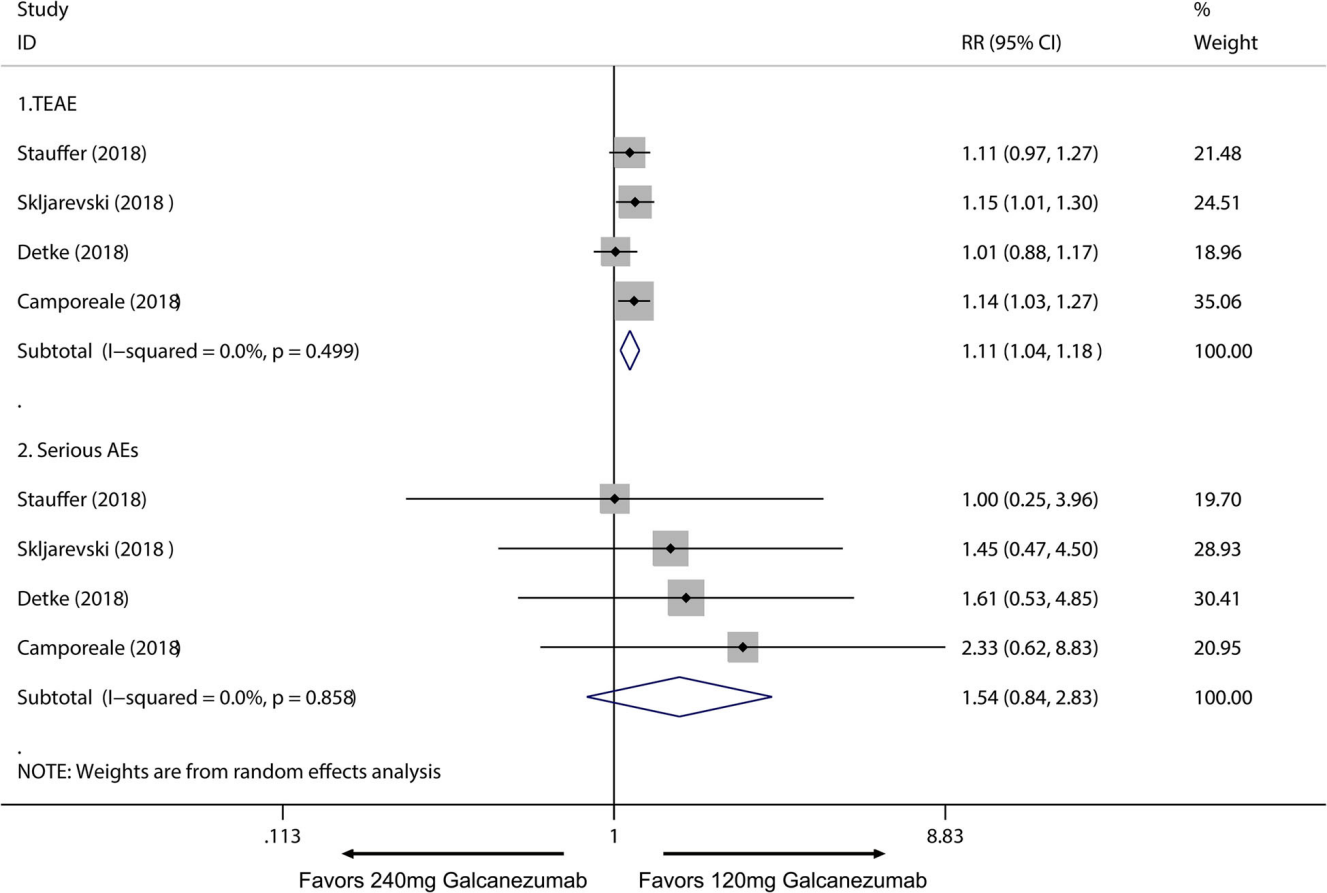
Comparison of SAEs between 120 mg and 240 mg galcanezumab. *RR* relative risk

**Fig. 10:**
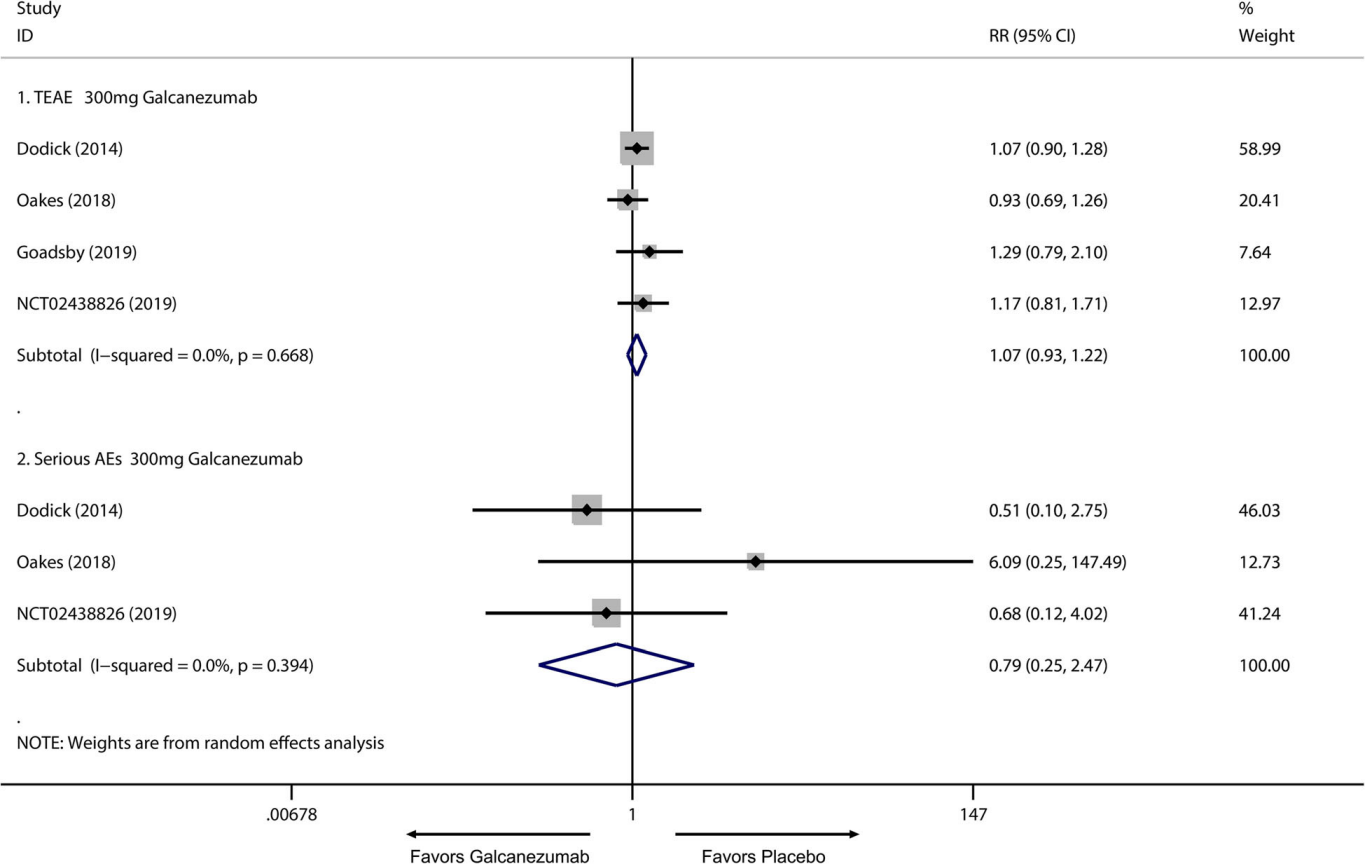
Pooled SAEs relative risk in 300 mg galcanezumab compared with placebo. *RR* relative risk

Then we plotted the discontinue rate for patients after receiving galcanezumab for 3 months, 6 months and 12 months, which demonstrates in the treatment groups, the discontinue rate was uniformly lower compared to placebo group, with an average 6 months discontinue rate of 14.24% (Table 3).

**Table 3:** Time dependent discontinue rate in treatment group compared with placebo

	3 month	6 month	12 month
Placebo	9.28%	16.78%	N/A
Treatment	5.74%	14.24%	22.22%

*N/A* Not Applicable

## Discussion

Our study is the first meta-analysis of the clinical use of galcanezumab in the treatment of migraine and cluster headache. It has revealed that the use of galcanezumab is related to a significantly reduced monthly headache frequency compared with placebo group with a relatively safe profile. Subsequently, for the does-response relationship, our data suggest that 120 mg galcanezumab per month is superior for treatment of migraine than 240 mg per month and 300 mg galcanezumab is effective for the treatment of episodic cluster headache with no significantly increased risk for adverse events.

We pooled 3889 subjects from 7 randomized clinical studies. First, we proved that 120 mg, 240 mg galcanezumab per month is effective for the treatment migraine when measured by response rate. Then, we analyzed the safety profile of galcanezumab for the treatment of migraine by comparing TEAEs and SAEs across different doses, which showed 240 mg group is related to increased TEAEs and SAEs compared with placebo group. To compare different doses on the treatment of migraine, we performed a subgroup analysis of 120 mg and 240 mg subgroup which demonstrated that 120 mg group has a significant lower rate of TEAEs compared with 240 mg group while has no significant difference on the reduction of headache frequency for the treatment of migraine, it further supports the use of galcanezumab in a dose of 120 mg per month. Moreover, we also demonstrated that 300 mg galcanezumab is effective against episodic cluster headache when measured by response rate and have no significantly increased risk for causing adverse events. Spontaneous relieving is observed in episodic cluster headache by previous studies [24], as a result, we chose 50% response rate at week 3 as our primary efficacy endpoint which is the secondary endpoint in the original research. One ongoing clinical trial of galcanezumab for the preventive treatment of chronic cluster headache was excluded from of study due to it didn’t reach its primary endpoint. Overall, it adds some evidence to the use of the novel drug type: CGRP monoclonal antibody in preventive therapy for episodic cluster headache and migraine.

As increased CGRP level is found to be highly correlated with headache attacks, it has becoming a promising target for headache relieving for decades. Direct CGRP receptor antagonists have been used for the acute attack management while CGRP monoclonal antibodies are been used on the preventive treatment. Previous studies have demonstrated the similarity on pathogenesis of migraine and cluster headache on pathogenesis and their close relationship with CGRP [25]. Currently proved CGRP monoclonal antibodies have great efficacy against migraine and episodic cluster headache. Efficacy of CGRP monoclonal antibodies has been surveyed by multiple clinical studies and meta-analysis [21]. Most of these studies has positive results regarding to its effectiveness in pain relieving.

Although Galcanezumab has a significant beneficial effect and is related to a lower risk for discontinue compared with placebo that is demonstrated by our research, the overall 100% response rate suggests the combination of subcutaneous infusion of galcanezumab with acute pain management is required to reach a superior clinical outcome. Thus, further studies are needed to reveal the overall outcome and potential interaction of combining galcanezumab with traditional therapies or direct CGRP receptor antagonists for the treatment of migraine and cluster headache.

Lack of adherence is one of the major problems in chronic disease prevention, this is also encountered by many other traditional preventive pharmacological treatments for migraine or cluster headache discontinue and self-changing of the drug is common in patient [26–29]. Discontinue can be related to bothersome adverse effects, high frequency of dosing or lack of efficacy. As we analyzed in our study, galcanezumab has a rather safe profile, with no mortality reported in all 7 clinical trials and no severe adverse event that is considered directly related to the use of the drug. One major advantage of galcanezumab is it only need to be administrated once monthly, this can be attributed to the long half-life of monoclonal antibodies. Furthermore, the incidence of discontinue is higher in placebo group than in the treatment group, which may contribute to the efficacy of the drug. Interestingly, in our pooled data, we observed a gradual increase in discontinue rate over time, which leads to a discontinue rate of 22.22% at 12 month which is still dramatically lower than 44% for traditional preventive therapy that was reported by previous retrospective cohort study [26]. However, because of the long disease course in these two diseases, further research should be undertaken to investigate patient adherence in a long-term setting.

Recent studies have not been restricted by testing galcanezumab for pain relieving on central nervous system, in 2018, Jin et al. conducted a randomized, placebo-controlled multi-center study investigating efficacy of galcanezumab on patients with knee osteoarthritis, however, it returns with negative result [30]. Because of the heterogeneity of the disease and lack of total adverse effect reported, we excluded this study form our meta-analysis.

One major limitation of our study is lack of enough clinical trials on cluster headache. The only included article for cluster headache targets at testing 300 mg galcanezumab for the treatment of episodic cluster headache [18], with a small sample size, caution must be applied for interpreting its clinical evidence. Plus, our study lakes the clinical data for chronic cluster headache trails. Another limitation for our study is lack of direct comparison of treating patients with galcanezumab versus previously used preventive therapies due to lack of large scaled RCTs. Lastly, our study is not able to gather enough data to demonstrate the time-response relationship of the drug due to lack of individual patient data.

## Conclusion

Use of galcanezumab for the treatment of primary headaches have a promising clinical application, it is related to a significantly reduced monthly headache frequency for the treatment of migraine and episodic cluster headache. As for the dose-response relationship, 120 mg has the same treatment efficacy with 240 mg group while related to a lower risk for adverse effects for the treatment of migraine. Thus, based on current studies, 120 mg per month is a preferred treatment method for migraine. Moreover, 300 mg per month galcanezumab is effective against episodic cluster headache that was proved by one randomized clinical trial and the use of 300 mg galcanezumab is not related to an increased risk for adverse events. Major limitation of this study is the lack of the clinical outcome of long-term use, thus, further studies are required to evaluate efficacy and safety in a long-term setting.

## Supplementary information

**Additional file 1: Supplement I**. ≥50%, ≥75% and 100% reduction in baseline monthly headache days for galcanezumab versus placebo in the treatment of migraine. **Supplement II**. Sensitivity analysis of ≥50% reduction in baseline monthly headache days for galcanezumab versus placebo showed that all of the consolidated results were stable. **Supplement III**. ≥75% reduction in baseline monthly headache days for 120 mg and 240 mg galcanezumab versus placebo. **Supplement IV**. 100% reduction in baseline monthly headache days for 120 mg and 240 mg galcanezumab versus placebo. **Supplement V**. ≥75% reduction in baseline monthly headache days for 120 mg galcanezumab versus 240 mg galcanezumab. **Supplement VI**. 100% reduction in baseline monthly headache days for 120 mg galcanezumab versus 240 mg galcanezumab. **Supplement VII**. Sensitivity analysis of ≥50% reduction in baseline monthly headache days for 120 mg galcanezumab versus 240 mg galcanezumab showed that all of the consolidated results were stable.

## Data Availability

All data generated or analyzed during this study are included in this review.

## Abbreviations

CGRP: Calcitonin gene-related peptide
CI: Confidence intervals
PRISMA: Preferred reporting items for systematic reviews and meta-analyses
RCTs: Randomized clinical trials
RR: Relative risk
SAE: Serious adverse events
TEAE: Treatment-emergent adverse events
WMD: Weighted mean differences
