# Supplementary material for: Different doses of galcanezumab versus placebo in patients with migraine and cluster headache: a meta-analysis of randomized controlled trials
Source: J Headache Pain. 2020 Feb 11;21(1):14. doi: 10.1186/s10194-020-1085-x (PMC7014619; doi:10.1186/s10194-020-1085-x)
Supplement: Supplementary file 1 — Additional file 1: Supplement I. ≥50%, ≥75% and 100% reduction in baseline monthly headache days for galcanezumab versus placebo in the treatment of migraine. Supplement II. Sensitivity analysis of ≥50% reduction in baseline monthly headache days for galcanezumab versus placebo showed that all of the consolidated results were stable. Supplement III. ≥75% reduction in baseline monthly headache days for 120 mg and 240 mg galcanezumab versus placebo. Supplement IV. 100% reduction in baseline monthly headache days for 120 mg and 240 mg galcanezumab versus placebo. Supplement V. ≥75% reduction in baseline monthly headache days for 120 mg galcanezumab versus 240 mg galcanezumab. Supplement VI. 100% reduction in baseline monthly headache days for 120 mg galcanezumab versus 240 mg galcanezumab. Supplement VII. Sensitivity analysis of ≥50% reduction in baseline monthly headache days for 120 mg galcanezumab versus 240 mg galcanezumab showed that all of the consolidated results were stable. [file 10194_2020_1085_MOESM1_ESM.docx]

**Title:** **Different doses of galcanezumab versus placebo in patients with migraine and cluster headache: a meta-analysis of randomized controlled trials.**

Yanbo Yang ^1,#^, Zilan Wang ^1,#^, Bixi Gao ^1^, He Xuan^2^, Yun Zhu ^1^, Zhouqing Chen ^1,*^, Zhong Wang ^1,*^

**Supplement Ⅰ**

**≥50%, ≥75% and 100%** **reduction** **in baseline monthly headache days for galcanezumab versus placebo in the treatment of migraine**

**
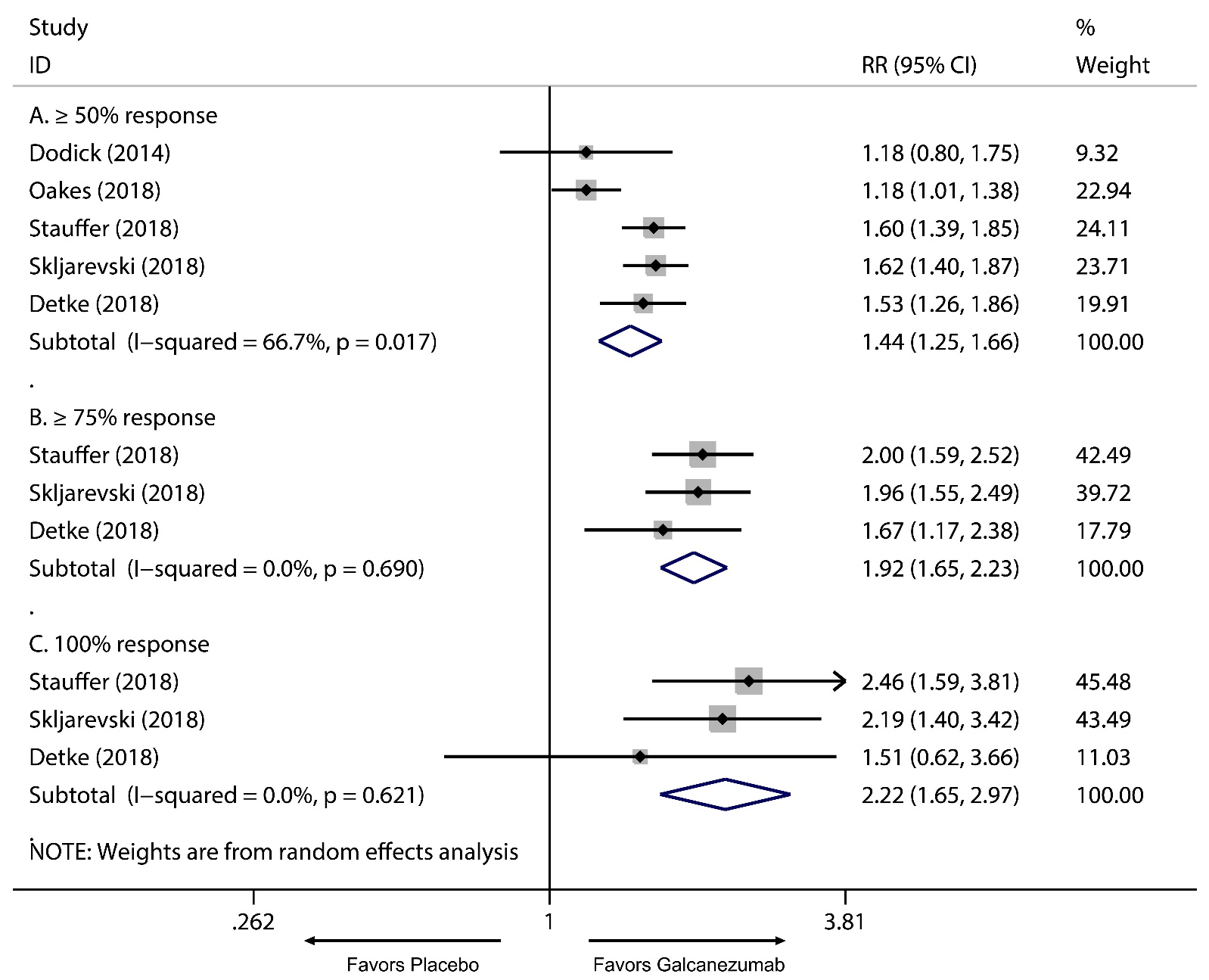
The diamond indicates the estimated hazard ratio (95% confidence interval) for all patients together.**

**Supplement Ⅱ**

**Sensitivity analysis of** **≥50% reduction in baseline monthly headache days for galcanezumab versus placebo showed that all of the consolidated results were stable.**

**
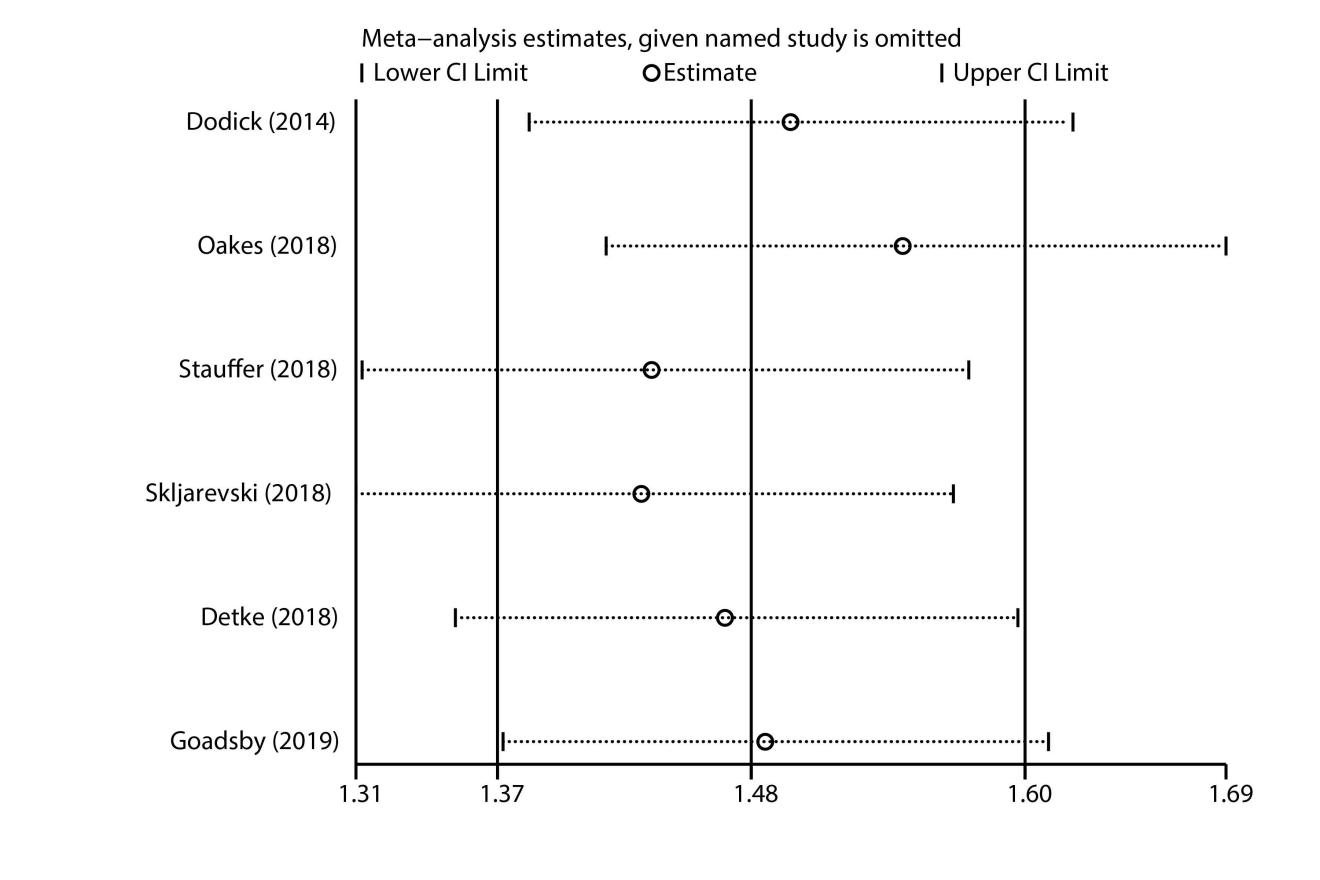
**

**Supplement Ⅲ**

**
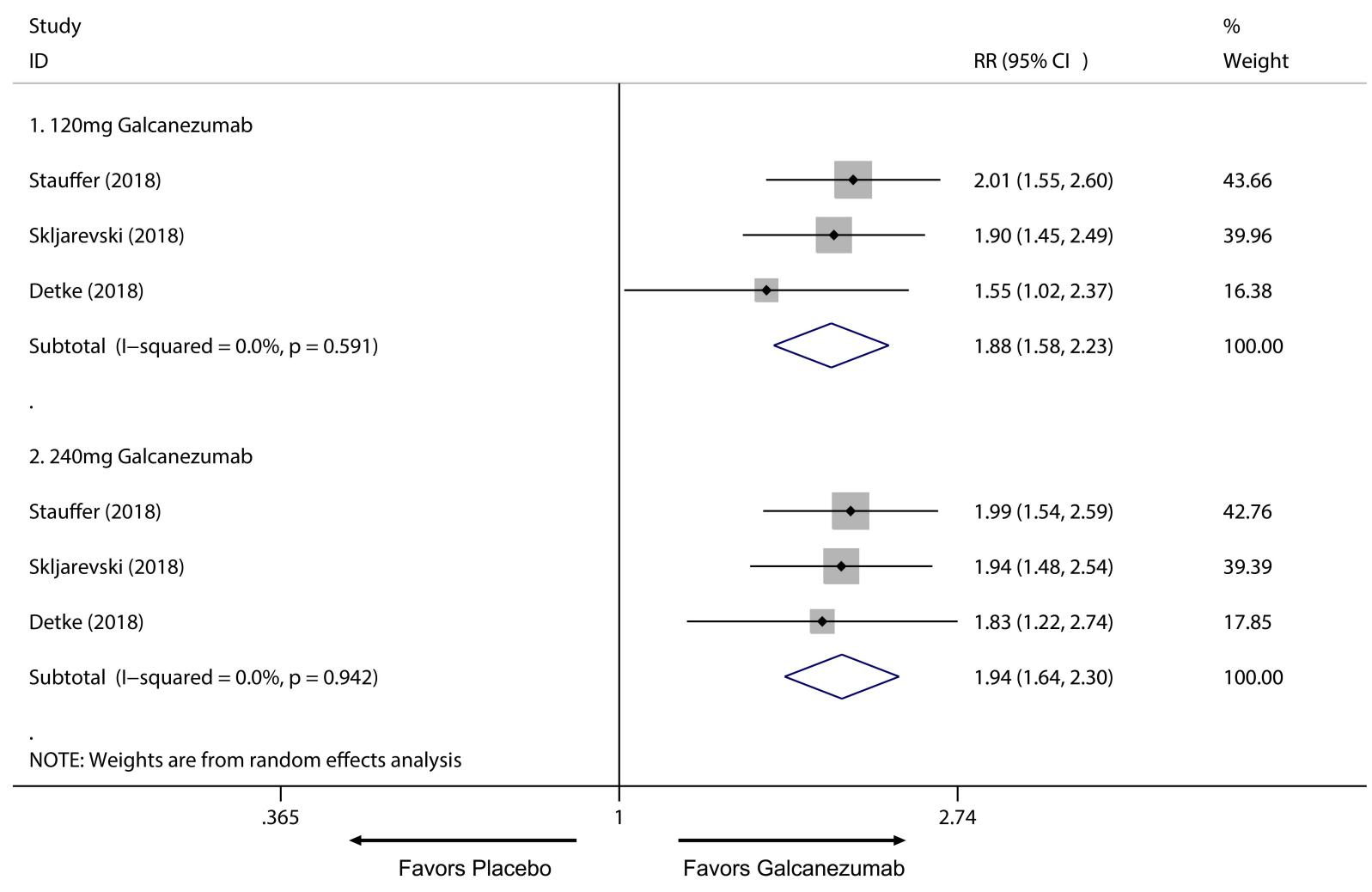
≥75% reduction in baseline monthly headache days for 120mg and 240mg galcanezumab versus placebo**

**Supplement Ⅳ**

**
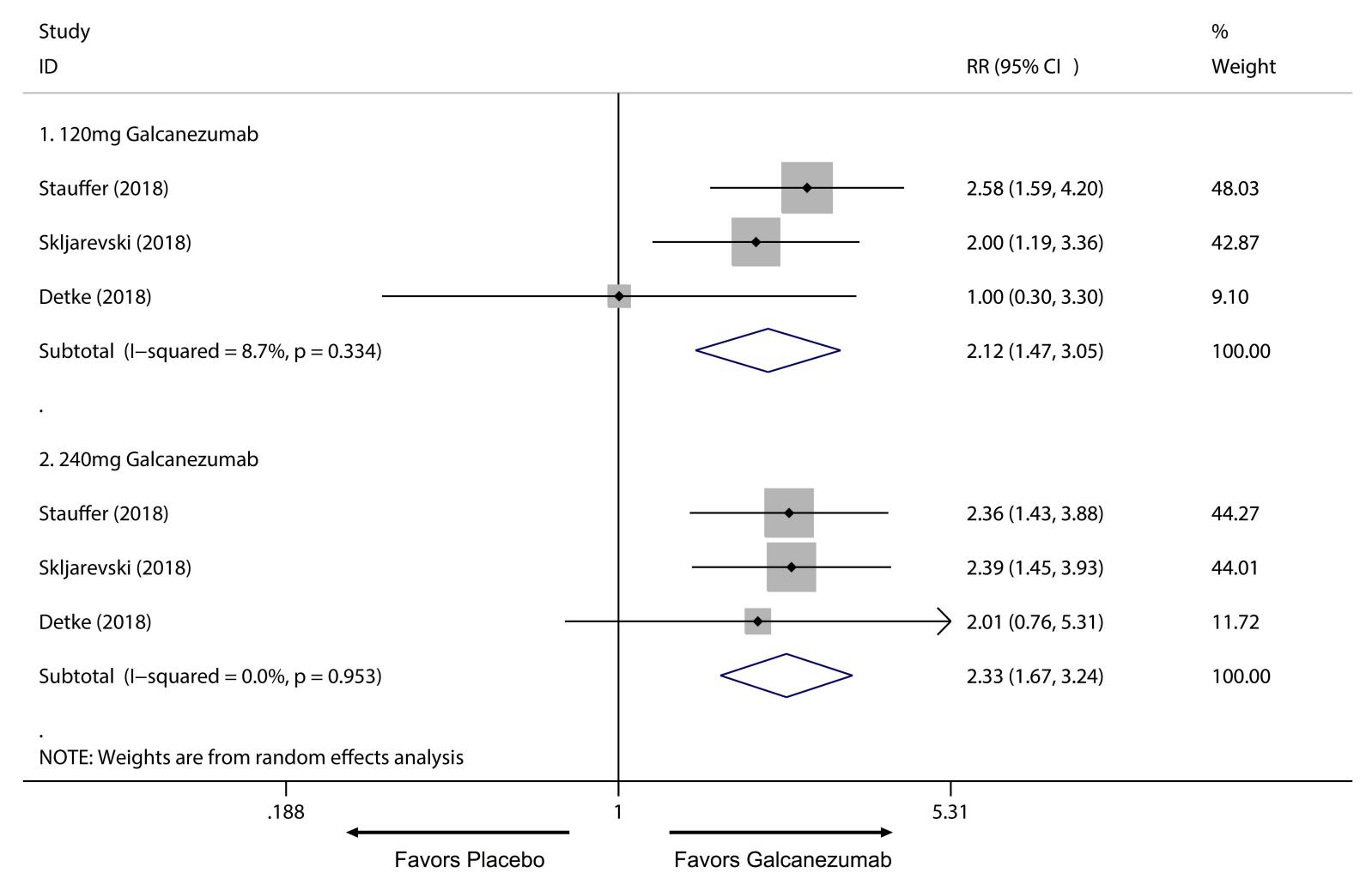
100% reduction in baseline monthly** **headache days for 120mg and 240mg galcanezumab versus placebo**

**Supplement Ⅴ**

**≥75% reduction in baseline monthly headache days for 120mg galcanezumab versus 240mg galcanezumab**

**
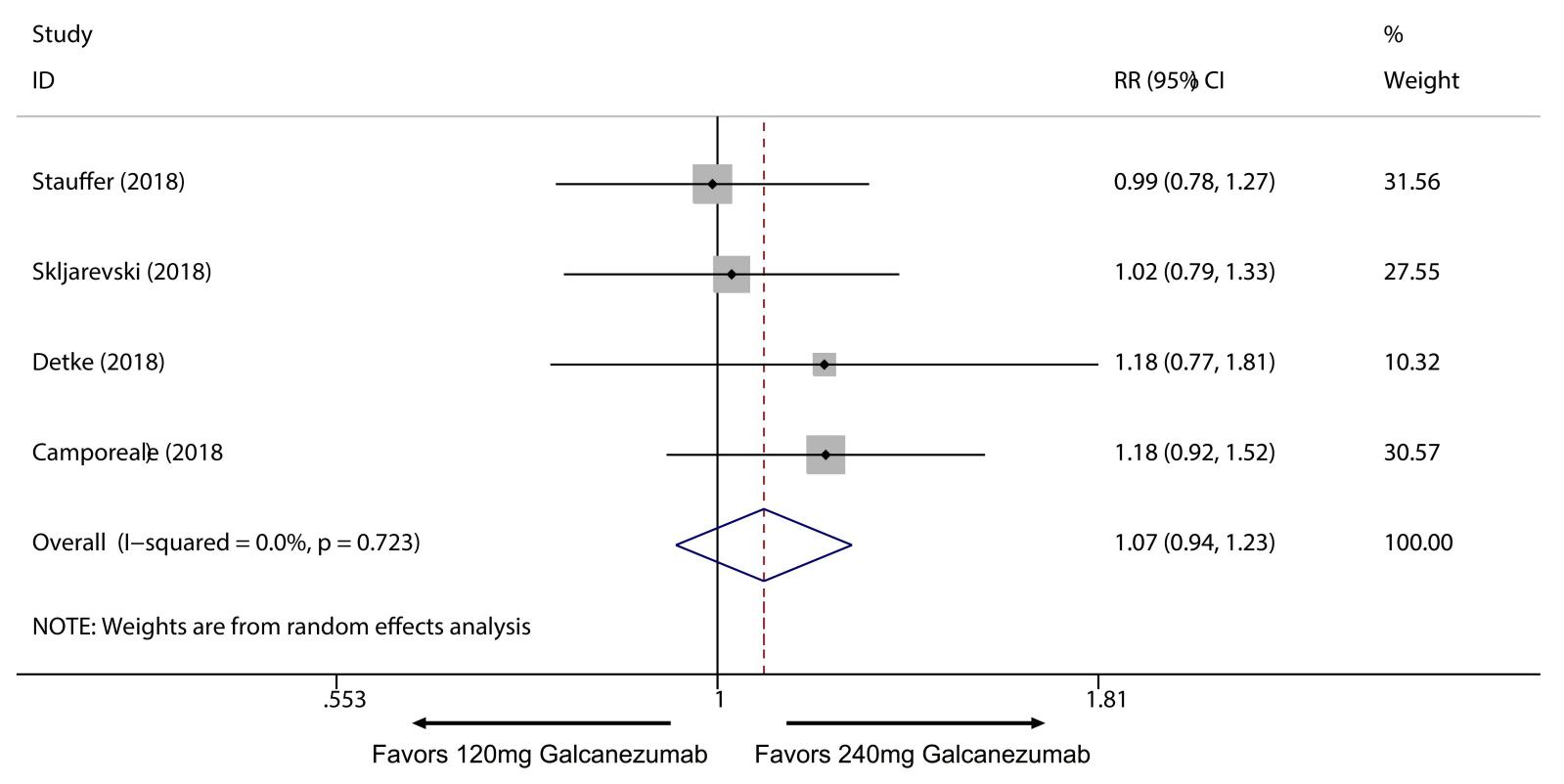
**

**Supplement Ⅵ**

**100% reduction in baseline monthly headache days for 120mg galcanezumab versus 240mg galcanezumab**

**
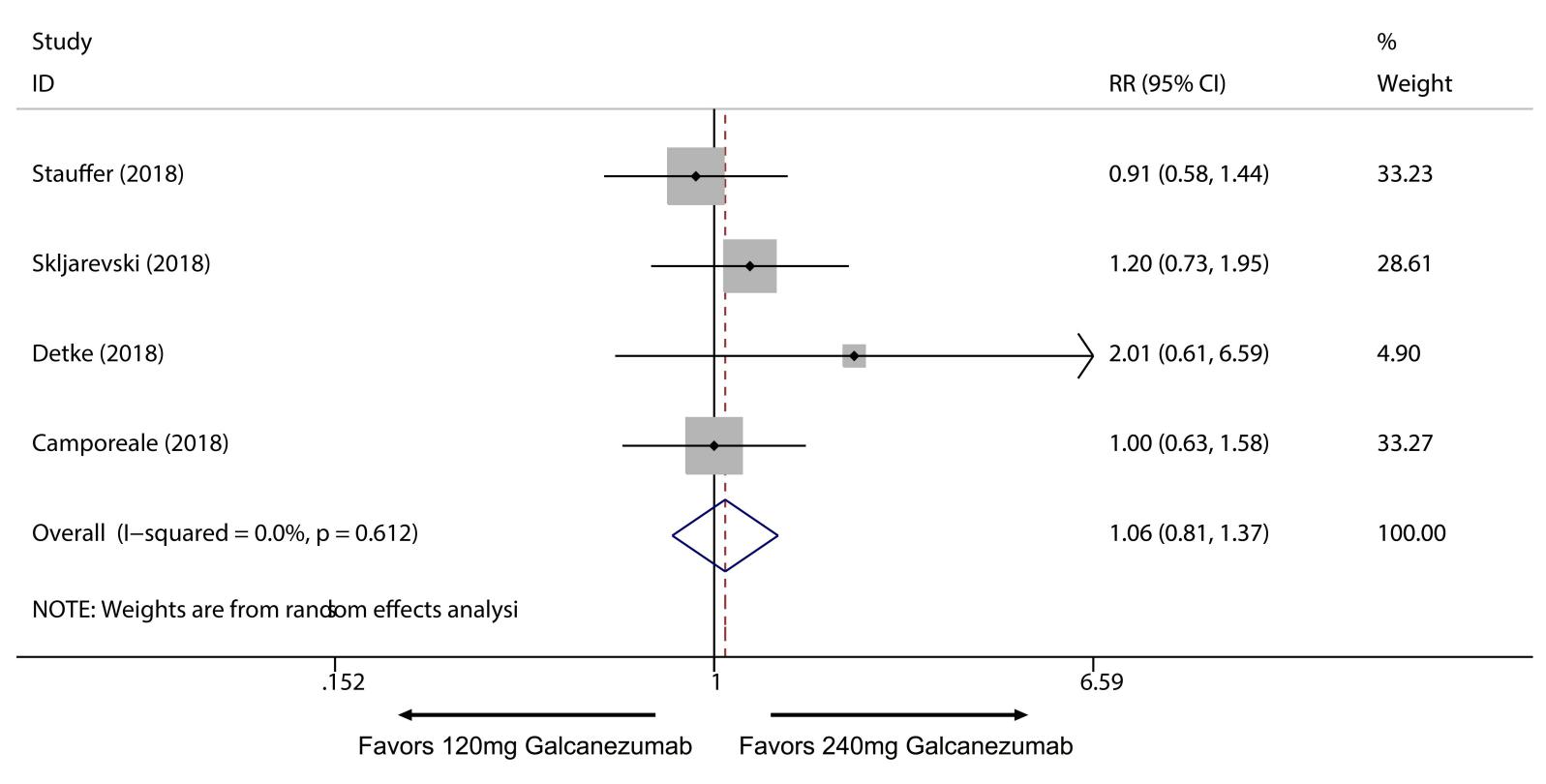
**

**Supplement Ⅶ**

**Sensitivity analysis of ≥50% reduction in baseline monthly headache days for 120mg** **galcanezumab versus 240mg galcanezumab showed that all of the consolidated results were stable.**

**
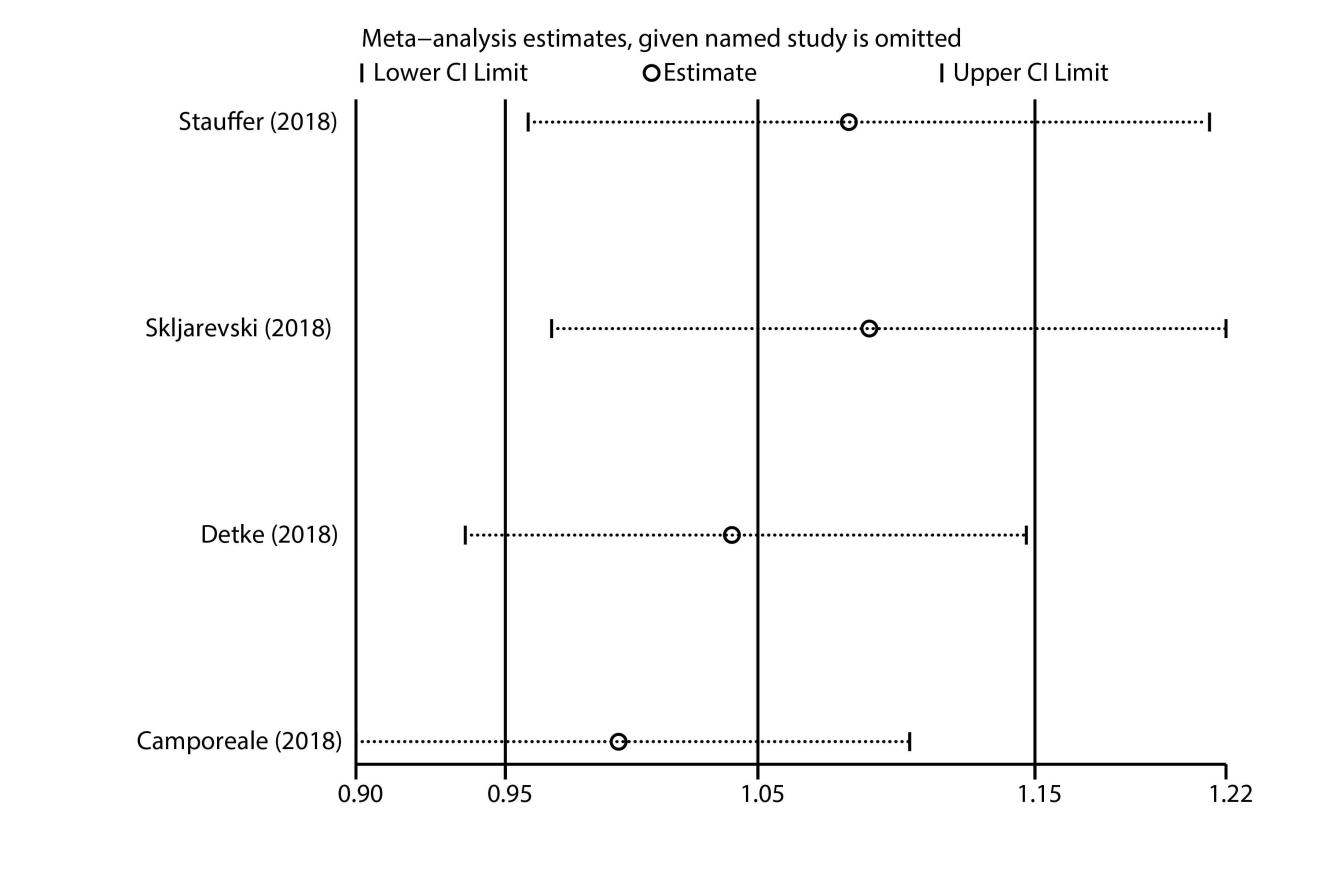
**
